# A theoretic predictive model to simulate the interference effects of acute aerobic exercise on rate of force development in weightlifters

**DOI:** 10.3389/fphys.2026.1785061

**Published:** 2026-05-08

**Authors:** Emidio E. Pistilli, Alan D. Mizener, Stuart A. Clayton

**Affiliations:** 1 Division of Exercise Physiology, Department of Human Performance, West Virginia University, Morgantown, WV, United States; 2 Cancer Institute, MD/PhD Training Program, West Virginia University, Morgantown, WV, United States

**Keywords:** aerobic interference, concurrent training, rate of force development (RFD), simulation, weightlifting

## Abstract

**Introduction:**

Compelling data supports the concept that concurrent strength and aerobic training can interfere with adaptations to strength training related to speed, power, and rate of force development (RFD). Studies on this topic have primarily utilized non-athlete participants, with data subsequently extrapolated to athlete populations. Since there may be hesitancy to have athletes involved in research on the interference effects of aerobic exercise on strength specific adaptations, we built a model to simulate these effects and developed an equation to predict interference. The hypothesis tested in this project was that aerobic exercise is the main driver of interference on weightlifting-associated RFD, and that this interference can be modeled to generate an equation to predict changes to RFD.

**Methods:**

Python software was used to generate the model, perform the simulation, and optimize variables that contribute to the interference effect. A theoretical nonlinear exponential interference model was created that simulates the changes in RFD from a force-time curve in response to acute aerobic exercise lasting from 2-minutes to 60-minutes in duration.

**Results:**

RFD sensitivity to aerobic exercise was modeled such that maximal RFD values were predicted to be reduced by approximately 70% after aerobic exercise less than 10 minutes, with further predicted reductions of 80% with durations greater than 10-minutes.

**Discussion:**

The prediction equation includes variables that can be adjusted by coaches, such as the rate of decay and the RFD scaling factor, to predict the interference of acute aerobic exercise on RFD in strength athletes. This would theoretically allow the coach to make informed decisions on training program design if there is a need to include aerobic exercise in a strength and/or power-based periodized plan. The simulation model described herein is theoretical and has not yet been empirically validated.

## Introduction

Performing both strength and aerobic exercise training (i.e., concurrent training) is recommended to develop and maintain musculoskeletal and cardiorespiratory fitness, respectively, in healthy adults (Garber et al., 2011). The expected training-induced adaptations from structured strength training and aerobic exercise training, when performed independently, are well-established and are divergent in nature as described in classic publications (Saltin, 1969; Pollock, 1973; Atha, 1981) and in more recent reviews (Jones and Carter, 2012; Grgic et al., 2018; Zouita et al., 2023). A recent review also summarizes the adaptations in the neuromuscular system in response to resistance training, including mechanisms associated with muscle fiber recruitment and firing frequency, neuromuscular junction function and neurotransmitter release patters, motoneuron excitability, motor cortex facilitation, and synaptic inputs (Lecce et al., 2026). The concept of an “interference” on strength training-induced adaptations when performing concurrent strength and aerobic exercise was first suggested by Hickson (1980) and has received significant research attention since this initial study. Published research on this topic suggests that concurrent strength and aerobic training may induce differing amounts of interference on training-associated adaptations in muscle strength and hypertrophy, depending on training volume, intensity, exercise order, training frequency and the exercising population (Dudley and Djamil, 1985; Dudley and Fleck, 1987; Callister et al., 1988; Hakkinen et al., 2003; Glowacki et al., 2004; Wilson et al., 2012; Sedano et al., 2013; Lee et al., 2020). However, the potential for interference from aerobic exercise on muscle hypertrophy and strength does become greater in athlete populations, especially for athletes involved in strength sports (Kraemer et al., 1995; Schoenfeld, 2021). Compelling data also exist suggesting that concurrent strength and aerobic training can interfere with specific adaptations to strength training related to speed, power, and rate of force development (RFD), variables that are associated with optimal nervous system contributions to performance (Dudley and Djamil, 1985; Dudley and Fleck, 1987; Hakkinen et al., 2003; Del Vecchio et al., 2022). When considering the collective adaptations expected from strength training, RFD is likely more sensitive to the inclusion of aerobic exercise than is muscle strength and hypertrophy.

The training programs of weightlifters focus almost exclusively on the competition lifts (i.e., snatch; clean and jerk) and weightlifting derivatives to enhance power and RFD (Suchomel and Sole, 2017; Suchomel et al., 2020; James et al., 2022; Takei et al., 2024), and rarely include periods of aerobic exercise. Recent publications also present compelling data on the effects of ballistic training (Lecce et al., 2025b) and maximal intended contraction velocity training (Lecce et al., 2025a) on strength, power and RFD in elite athletes. To the Authors’ knowledge, no study has been completed in highly trained weightlifters to determine the potential interference effect of acute aerobic exercise on adaptations to weightlifting training and performance. Published studies on this topic utilize healthy subjects that are typically not training for sport performance and conclusions are subsequently extrapolated to athlete populations (Dudley and Djamil, 1985; Kraemer et al., 1995; Hakkinen et al., 2003; Glowacki et al., 2004; Lee et al., 2020). Given that there may be hesitation to have weightlifters involved in a study to directly determine the interference effects of aerobic exercise on performance, there is a need for an alternative approach to predict or estimate these interference effects. The goal of this project was to create a theoretical predictive model to simulate the effects of varying durations of acute aerobic exercise on the subsequent changes to RFD in weightlifters, and to utilize the model to generate an equation that can predict the changes in RFD immediately following acute aerobic exercise; not to experimentally verify interference. The hypothesis tested in this project was that aerobic exercise is the main driver of interference on weightlifting-associated RFD, and that this interference can be modeled to generate an equation to predict changes in RFD. We built the model such that acute aerobic exercise will cause the slope of a force-time curve from an isometric mid-thigh pull to decrease (i.e., flatten) with increasing durations of acute aerobic exercise. The RFD prediction equation which we have generated allows a weightlifting coach to enter an athlete’s current RFD value, and then estimate the change in RFD, in both absolute loss and percentage loss, following any duration of aerobic exercise. The value in this equation is that it will allow a coach to make informed decisions on acute program design and estimate the potential effects to RFD if aerobic exercise of any duration is performed.

## Methods

### Experimental approach to the problem

The purpose of this project was to develop a predictive equation that can be utilized by coaches and athletes to predict the interference effects of acute aerobic exercise on the force-time curve in weightlifters, specifically predicting changes in RFD that are reflected in the initial slope of the force-time curve. Code to run the simulations was written in Python (version 3.13.7). A model was created to simulate the effects of increasing durations of aerobic exercise on the force-time curve, from a minimum of 2-minutes to a maximum of 60-minutes of aerobic exercise. The model makes the following assumptions: 1) aerobic exercise duration, defined as “d”, is the main driver of interference on RFD; 2) RFD is sensitive to aerobic exercise such that negative effects can be observed after as little as 2 minutes of aerobic exercise; 3) the rate of RFD declines nonlinearly with increasing durations of aerobic exercise such that greater RFD loss is seen with aerobic exercise lasting <10 minutes and continued smaller reductions in RFD occur with aerobic exercise >10 minutes; 4) RFD acquired from an isometric mid-thigh pull test is associated with weightlifting performance (Beckham et al., 2013; Rochau et al., 2024; Soriano et al., 2025); 5) while differences in absolute RFD from an isometric mid-thigh pull exist based on sex and weightlifting experience (Haff et al., 2005; Hornsby et al., 2017; Suarez et al., 2019), maximum RFD in the model is set to 15,000 N to represent the conceptual interference from aerobic exercise. The theoretical predictive model applies to acute aerobic exercise in the form of running and it may not sufficiently distinguish the effects of differing aerobic exercise modalities. Furthermore, the predictive model has not been validated with empirical data, and it is not intended to model exercise recovery kinetics.

### Simulation variables and model generation.

**Figure 1:**
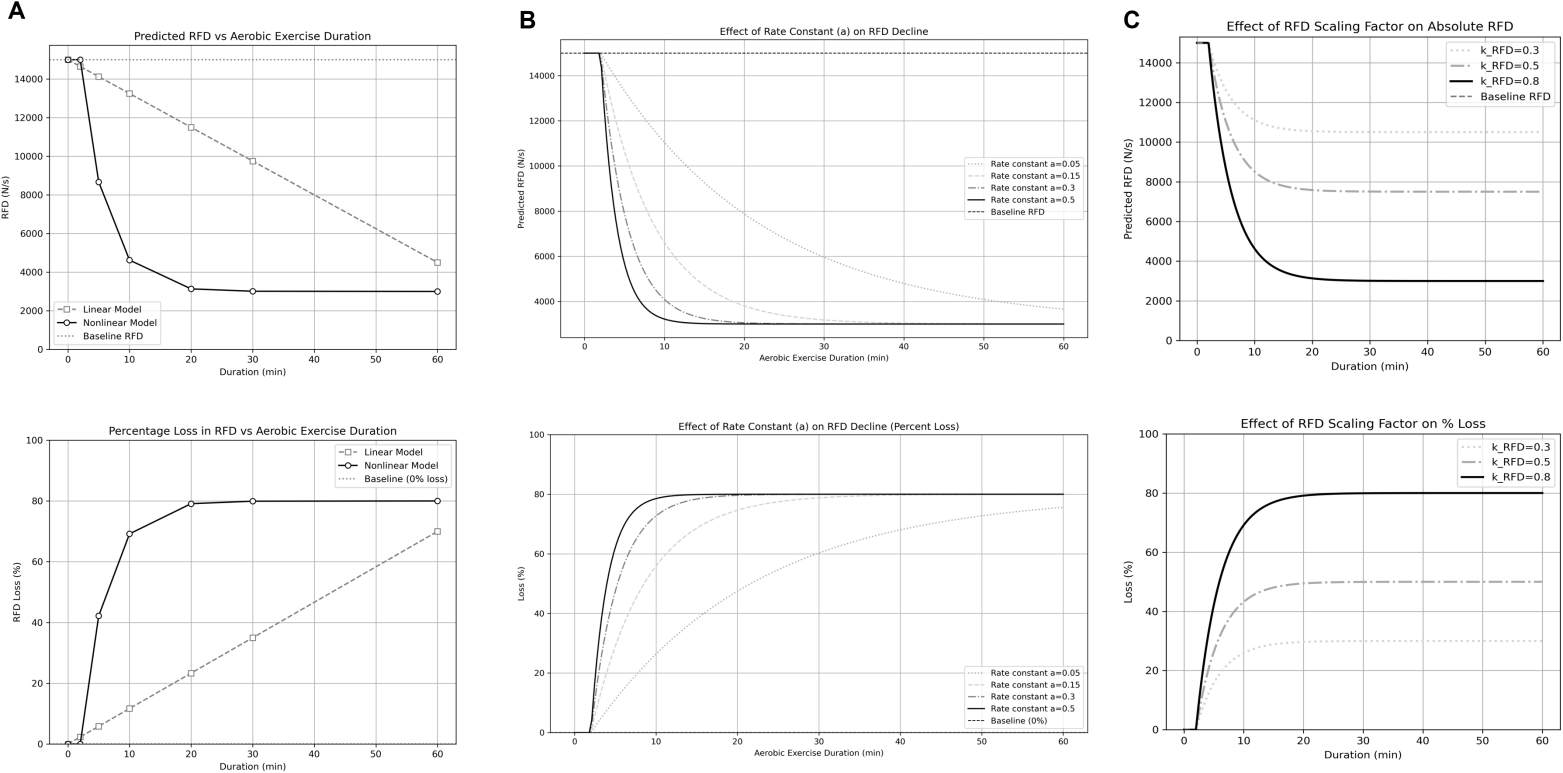
Simulation variables and model generation. **(A)** Comparison of linear and nonlinear loss of RFD in response to aerobic exercise. The initial step in developing this model was to determine whether the loss of RFD following acute aerobic exercise would occur as a progressive linear loss or an exponential nonlinear loss. The nonlinear loss of absolute RFD produced a curve depicting a rapid decline in response to aerobic exercise <10-minutes in duration, with predicted absolute RFD loss plateauing after 20-minutes of aerobic exercise. The nonlinear loss of RFD percentage produced a curve that predicted 70% loss in response to 10-minutes of aerobic exercise, with percentage loss approaching 80% after 20-minutes of aerobic exercise. **(B)** The effects of differing rates of decay on the loss of RFD in response to acute aerobic exercise. The rate of decay was adjusted to produce a series of curves depicting the predicted absolute loss and percent loss of RFD in response to acute aerobic exercise. A value of 0.3 was selected to represent the expected sensitivity of RFD to acute aerobic exercise in weightlifters. **(C)** The effects of differing RFD scaling factors on the loss of RFD in response to acute aerobic exercise. The RFD scaling factor was adjusted to produce a series of curves depicting the absolute loss and percent loss of RFD following acute aerobic exercise. A value of 0.8 was selected to represent the theoretical maximum loss of RFD, which would be equivalent to a loss of 80% of an athlete’s maximal RFD.

Specific variables used to generate the predictive model are defined in **Table 1**, including default values for these specific variables. The effect of aerobic exercise duration on RFD was initially modeled with either a linear or nonlinear rate of decline. **Figure 1A** displays the general form of predicted absolute and percentage RFD loss when modeled as a linear rate of decay compared to a nonlinear rate of decay. The nonlinear rate of decay model was selected based on the predicted effects of aerobic exercise on the nervous system and sensitivity of RFD to this mode of exercise in weightlifters. As such, greater loss of RFD is seen with aerobic exercise <10-minutes, with continued smaller changes observed with durations of aerobic exercise >10-minutes. The nonlinear rate of decay was subsequently modeled by adjusting “α” to simulate the sensitivity of RFD to the effects of aerobic exercise (**Figure 1B**). Lastly, the nonlinear RFD scaling factor was modeled by adjusting “κ” to establish the maximal limit for RFD loss (**Figure 1C**). Based on these simulations, a nonlinear model was selected with a rate of decay (α) set at a value of 0.3 and an RFD scaling factor (κ) set at 0.8, which would be applicable to weightlifters and other anaerobic athletes training for enhanced RFD.

**Table 1:** Variables used to generate the predictive model.

Parameter	Definition	Default value
RFD_0_	Baseline peak rate of force development (N.s-1)	15,000
*d*	Duration of aerobic exercise (minutes)	0-60
κ_RFD_	Scaling factor; establishes maximal reduction in RFD	0.8
α	Rate constant; sets the steepness of the predicted RFD curve (i.e., how quickly interference occurs)	0.3
*d_0_*	Time at which RFD is predicted to begin declining (minutes)	2
*(d – d_0_)*	Positive-part operator; equals 0 when *d* < *d*_0_	Na

The general form of the prediction equation is as follows:


$$RFD_{aerobic}(d)=RFD_{0}\times \left[1-k_{RFD}\cdot (1-e^{-a_{RFD}\cdot (d-d_{0,RFD})+})\right]$$

Using the variable default values from **Table 1**, the prediction equation would be calculated as follows:


$$RFD_{aerobic}(d)=15000\times \left[1-0.8\cdot (1-e^{-0.3\cdot (10-2)+})\right]$$

$$RFD_{aerobic}(d)=4089N\cdot s^{-1}$$

### Prediction equation code and web application

The RFD prediction equation was further developed into a code that allows coaches to input an athlete’s known peak RFD value and the duration of aerobic exercise to estimate the absolute and percent change in RFD. The resulting RFD value represents the change in slope of the initial phase of the force-time curve when an isometric mid-thigh pull is performed subsequent to the single bout of aerobic exercise. The full interactive code to allow coaches to predict changes in RFD values after aerobic exercise is freely available at https://github.com/PistilliLab/RFDsimulator. In addition, an interactive web application has been created that allows a coach to enter the above parameters and estimate RFD changes after aerobic exercise (https://rfdsimulator.streamlit.app/). This web application will allow coaches to enter baseline RFD values and aerobic exercise durations as well as adjust the rate of decay and RFD scaling factor to customize the prediction equation for specific athlete populations.

### Ethics approval

This study did not require Institutional Review Board (IRB) approval as it does not meet the definition of human subjects research. The model presented is purely theoretical and predictive, and was developed using simulated parameters to predict potential interference effects of aerobic exercise on rate of force development. No experiments, interventions, observations, or data collection involving human participants were conducted.

## Results

### Model simulation

**Figure 2:**
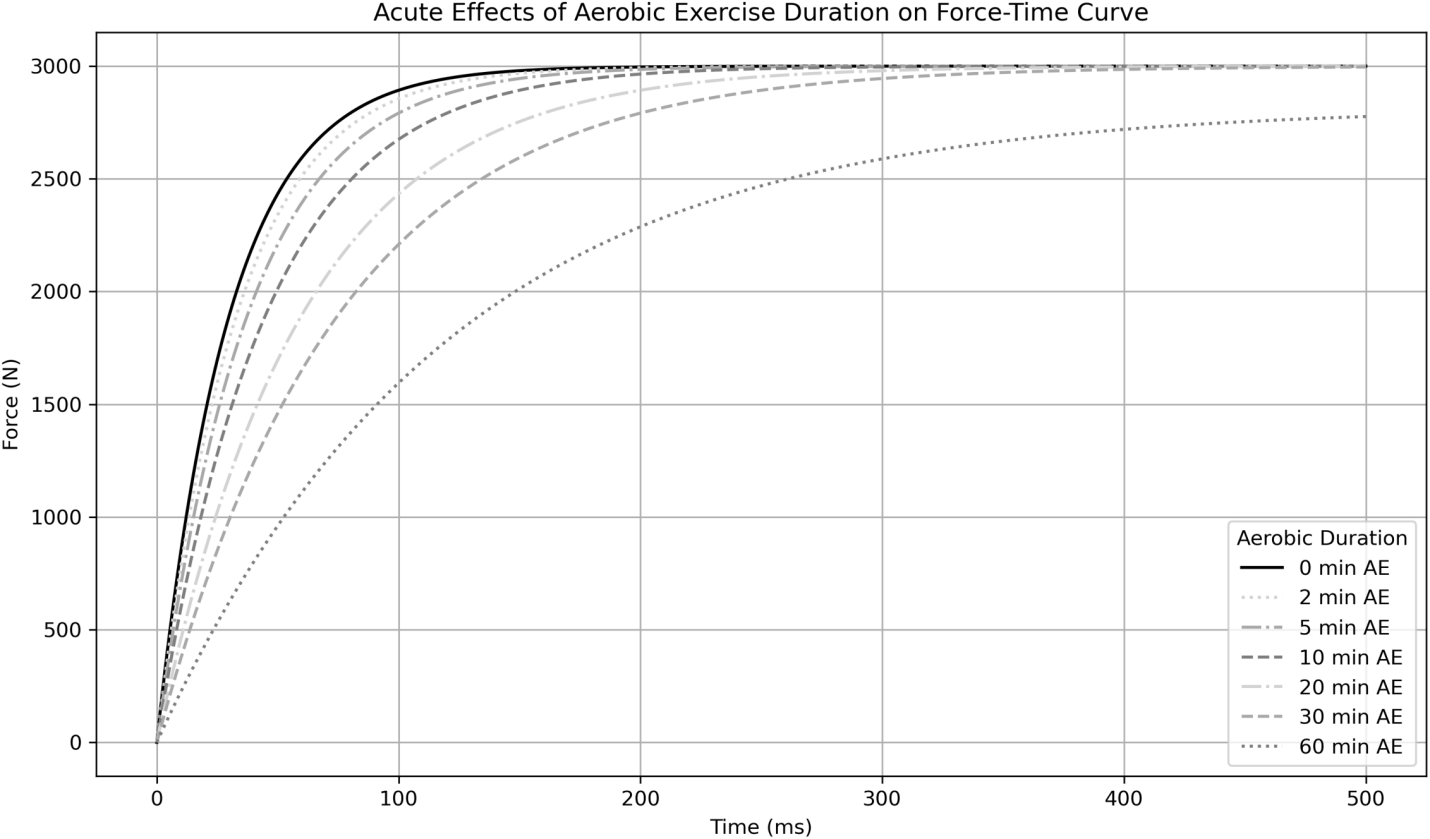
The effects of acute aerobic exercise on the force-time curve. The simulation model was used to estimate the changes in the force-time curve produced from an isometric mid-thigh pull following various durations of aerobic exercise. A series of force-time curves were generated in response to increasing duration of aerobic exercise, from 5-minutes to 60-minutes, and can be compared to the force-time curve following no aerobic exercise (solid line). The changes in the force-time curves were produced using a rate of decay value of 0.3 and an RFD scaling factor of 0.8.

**Figure 3:**
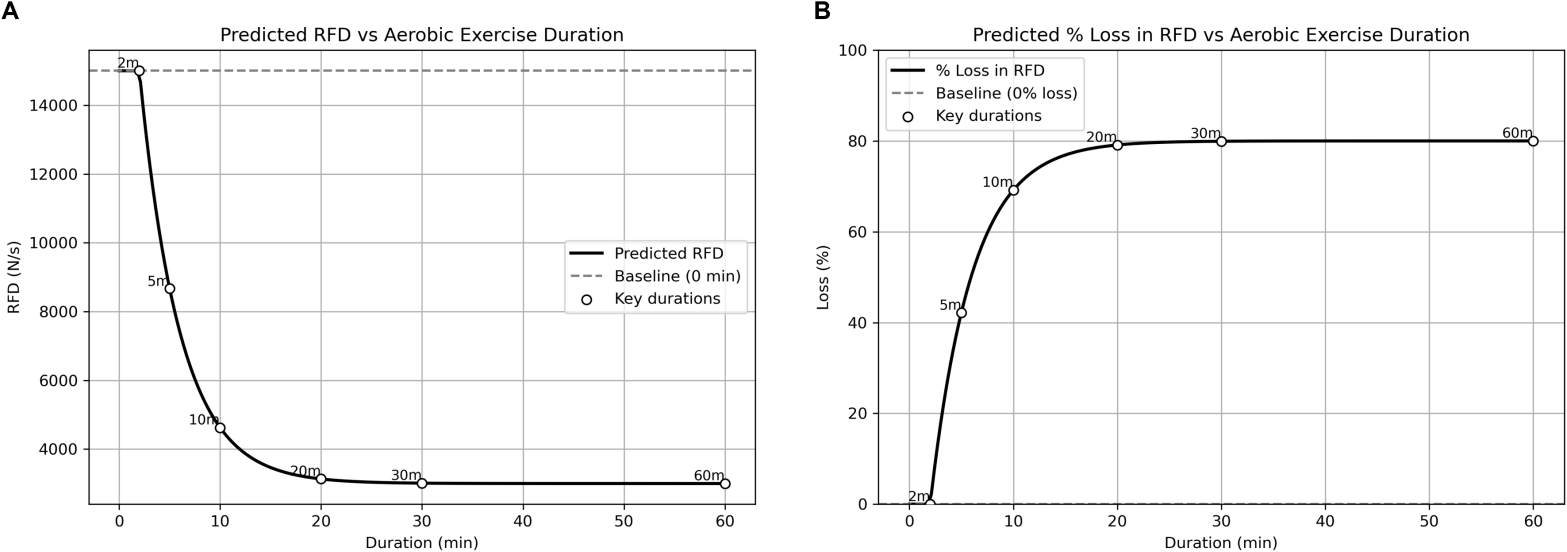
The final model depicting the estimated loss of RFD in response to various durations of acute aerobic exercise. The final model parameters used to generate these curves were a rate of decay value of 0.3, an RFD scaling factor of 0.8, and the assumption that RFD loss would be minimal following aerobic exercise <2-minutes. **(A)** The absolute loss of RFD in response to acute aerobic exercise. The rapid initial loss of RFD occurs in response to aerobic exercise durations <10-minutes, with smaller losses that may plateau following aerobic exercise >20-minutes. **(B)** The percentage loss of RFD in response to acute aerobic exercise. The rapid initial loss of RFD approaches 70% in response to aerobic exercise durations <10-minutes, with smaller losses that approaching 80% following aerobic exercise >20-minutes.

The nonlinear RFD interference model was tested with aerobic exercise durations in minutes set at: 0; 2; 5; 10; 20; 30; and 60. The simulation produced a series of force-time curves that represent an isometric mid-thigh pull after completing the different durations of aerobic exercise (**Figure 2**). Absolute RFD loss and the percentage RFD loss was subsequently predicted from these force-time curves based on an initial maximum value for RFD of 15,000 N.s^-1^, a rate of decay of 0.30, and a maximal RFD scaling factor of 0.8. **Figure 3A** shows the predicted changes in absolute RFD with increasing durations of aerobic exercise, such that RFD is predicted to drop after more than 2-minutes of aerobic exercise. Furthermore, the steep portion of the predicted absolute RFD loss curve occurs with <10-minutes of aerobic exercise, with smaller and less dramatic changes with >10-minutes of aerobic exercise, reflecting the sensitivity of RFD to aerobic exercise. **Figure 3B** shows the predicted percentage loss of RFD with increasing durations of aerobic exercise. The steep portion of the predicted percentage loss of RFD curve occurs with <10-minutes of aerobic exercise, such that approximately 60-70% of baseline RFD is predicted to be lost. With aerobic exercise >10-minutes, the predicted percentage loss of RFD continues to a maximum of ~80% of baseline RFD, based on the RFD scaling factor. Specific examples of the predicted absolute change and predicted percent loss of RFD following increasing durations of aerobic exercise are presented in **Table 2**; these values were generated through simulation, not using experimental data.

**Table 2:** Predicted absolute change and percent loss of RFD following aerobic exercise.

Aerobic duration (*d*)	Predicted RFD (N.s^-1^)	Predicted RFD loss (%)
0	15,000	0
2	15,000	0
5	7879	48%
10	4089	73%
20	3054	80%
30	3002	80%
60	3000	80%

### Coach tunable variables

The Python code allows a coach to adjust the variables based on the specific athletes that may be tested. After entering an athlete’s absolute RFD value, the following variables can be adjusted: aerobic exercise duration (d); rate of decay (a); and RFD scaling factor (k). The model produces a predicted RFD loss curve based on duration of aerobic exercise from 2-minutes to 60-minutes. A coach would be able to enter in any aerobic exercise duration within this range and receive an estimate of the interference on RFD, reflecting any situation in which an athlete may need to perform aerobic exercise (i.e., rehabilitation; conditioning; etc.). The rate of decay is an estimate of the sensitivity of RFD to aerobic exercise. Therefore, the coach can adjust the rate of decay to simulate changes to RFD in athletes that may be more or less susceptible to the interference of aerobic exercise (**Figure 1B**). Lastly, the RFD scaling factor can be adjusted to set the maximum predicted loss of RFD due to aerobic exercise interference to estimate these changes in numerous types of athletes (**Figure 1C**).

## Discussion

The basic principles of specificity and overload provide the framework for training program design. Strength & Conditioning Coaches utilize these two principles to manipulate training volume and intensity throughout the course of a program in order to peak athletes for competitions. This is the basis for the concept of Periodization, defined as a “theoretical and practical construct that allows for the systematic, sequential, and integrative programming of training interventions into mutually dependent periods of time in order to induce specific physiological adaptations that underpin performance outcomes” (Haff and Triplett, 2016). Training volume and intensity are manipulated in the training of athletes to enhance specific performance variables such as maximum force output, RFD, and power. RFD is specific to athletes that require maximal force output in very short periods of time, such as weightlifters (Garhammer, 1980; Stone et al., 2005; Storey and Smith, 2012; Suarez et al., 2019; Takei et al., 2024), is based on the ability of the nervous system to rapidly activate skeletal muscles and can be enhanced with specific training methods (Nardone et al., 1989; Hakkinen et al., 2003; Cormie et al., 2010; Cormie et al., 2011; Del Vecchio et al., 2022; James et al., 2022). A significant body of literature exists to support the concept that aerobic exercise may have a negative effect on aspects of nervous system function, which is tightly connected to RFD, power, and speed (Dudley and Djamil, 1985; Dudley and Fleck, 1987; Hakkinen et al., 2003; Del Vecchio et al., 2022). In addition, RFD acquired from an isometric mid-thigh pull test is associated with weightlifting performance, supporting our use of this test as a sensitive measure of the potential interference of aerobic exercise on RFD (Beckham et al., 2013; Rochau et al., 2024; Soriano et al., 2025). Therefore, athletes and coaches should be aware of this potential interference effect when designing training programs for athletes that require peak RFD and consider the risk-to-benefit ratio of including aerobic exercise in the training program for these athletes.

It is our assumption that coaches involved in the training of weightlifters would be hesitant to allow their athletes to be involved in a research study to directly test the interference effect of aerobic exercise on performance, which was the reason to conduct this predictive simulation. Since we did not utilize experimental data to generate this model, we can refer to a prior publication that described performance variables in weightlifters across 20 weeks of training (Hornsby et al., 2017). This study utilized active and competitive male and female national caliber weightlifters. Isometric mid-thigh pull tests were performed, as part of a laboratory test battery, during training weeks 1, 6, 10, 13, 17, and 20, associated with different phases of periodized training. At week 1, the male weightlifters had a group average RFD value of 16,652 N.s^-1^ and the female weightlifters had a group average RFD value of 7663 N.s^-1^. We can estimate the absolute RFD and percentage RFD loss in these groups using the new RFD interference model with the following variables: rate of decay set at 0.3; RFD scaling factor set at a maximum of 0.8; duration of aerobic exercise set at 10 minutes. In this example, the estimated RFD would be reduced by 73% following 10-minutes of aerobic exercise, which would be 4538.9 N.s^-1^ for the male weightlifters and 2088.7 N.s^-1^ for the female weightlifters.

The principle of training specificity is that training should be based on the movement patterns of athletes and that muscles involved in those movements should be targeted for strengthening. As stated by Gary Dudley in 1985, “obviously, the nature of the adaptative response to training is specific to the training stimulus” (Dudley and Djamil, 1985; Dudley and Fleck, 1987). In the study by Dudley and Djamil (1985), recreationally trained people were randomly assigned to 1 of 3 groups that performed exercise training for 7 weeks: an aerobic training only group; an isokinetic strength training only group; a concurrent training group that performed both of the aerobic and isokinetic strength training programs. At the end of the 7 weeks, there were no differences in improvement of aerobic power when comparing the aerobic training group and the concurrent training group, suggesting that concurrent aerobic and strength training does not interfere with improvements in aerobic capacity and subsequent studies have confirmed this observation (Kraemer et al., 1995; Hakkinen et al., 2003; Glowacki et al., 2004; Sedano et al., 2013; Lee et al., 2020). Participants in the isokinetic strength training group improved torque at all velocities tested that were equal to and below the velocity in which training occurred (i.e., 4.19 rad.sec^-1^). Interestingly, participants in the concurrent training group only improved torque at the three lowest velocities tested, while no improvements were observed at the velocity in which training occurred. Furthermore, Del Vecchio et al. (2022) demonstrated that training for maximal force production and fast contraction speeds are mutually exclusive and increases in RFD are due more to the ability to increase the recruitment speed of motor units than to the amount of muscle mass that is producing that force. Extending these published results to sport performance, it may be concluded that aerobic exercise in the training program of an anaerobic athlete has the potential to limit force production at fast velocities. This has obvious implications for specific athlete populations that require differing expressions of muscle strength to be successful and any athlete that needs to produce rapid muscle contractions against a resistance should limit exposure to aerobic exercise, as there is the potential to limit RFD and power adaptations to training. Given the numerous reports that demonstrate that aerobic exercise interferes with speed, power, and nervous system adaptations (Dudley and Djamil, 1985; Kraemer et al., 1995; Hakkinen et al., 2003; Glowacki et al., 2004; Lee et al., 2020), athletes that require high RFD such as weightlifters should perform as little aerobic exercise as possible. Reconsidering the mechanisms of aerobic exercise adaptations and how those may impact anaerobic training adaptations is important when strength coaches develop overall training programs for their athletes.

There are a few limitations to this study that should be acknowledged. First, the predictive model was not empirically validated, and parameters were not derived from experimental datasets. Also, individual athlete variability is not modeled, and the predicted 60-80% RFD reduction represents a theoretical projection. Second, the model was built on the assumptions identified in the methods section and therefore may not address situations outside of these *a priori* assumptions. For example, the rate of decay used in the model was selected based on our interpretation of the literature on the effects of aerobic exercise on nervous system function and the sensitivity of RFD to this mode of exercise. However, the rate of decay can be adjusted in the model based on each coach’s interpretation of this effect. Third, we hypothesized that aerobic exercise duration was the main driver of the interference on RFD. But we cannot determine how residual fatigue from acute aerobic exercise on other body systems may impact these RFD estimates. Additionally, we did not model the potential interference of specific types of aerobic exercise, such as running compared to cycling. Fourth, the interference estimated in this model reflects how the RFD may be affected when the isometric mid-thigh pull is performed relatively soon after acute aerobic exercise, within 1–2 hours. The model is not able to provide information on how soon RFD would be recovered after completing the acute aerobic exercise or how RFD would be affected with periods of chronic aerobic exercise. Lastly, while the theoretical predictive model is based on the assumption that aerobic exercise interferes with nervous system function related to RFD, this study cannot provide details on which mechanisms of nervous system function may be altered in response to acute aerobic exercise.

In conclusion, we were able to produce an equation that can predict interference of various durations of acute aerobic exercise on subsequent RFD from an isometric mid-thigh pull in weightlifters. This prediction equation and model is specific to weightlifters, but the variables included in the equation can be adjusted for other populations of athletes. The simulation model predicts that RFD may be negatively impacted after as little as 2-minutes of aerobic exercise and that 60%-70% of the maximal RFD may be lost after 10-minutes of aerobic exercise. However, any duration of aerobic exercise, up to 60-minutes, can be tested with this model. The prediction equation can be used by strength coaches to predict the interference of acute aerobic exercise on RFD and subsequently allow the coach to make informed decisions on training program design if there is a need to include aerobic exercise in a strength and/or power-based periodized plan. However, the model described herein represents theoretical predictions and empirical validation is required before practical implementation by strength coaches.

## Conclusions and practical applications

Coaches that work with weightlifters should consider the tissue and system-level adaptations associated with chronic aerobic and strength training and the potential for aerobic exercise to interference with certain performance variables. Generally, it is recommended to limit aerobic exercise in the overall training program of weightlifters and possibly other anaerobic athletes that train for RFD. While every athlete is different and the performance needs of sports vary, the general conclusion of published studies suggests that aerobic exercise may induce sub-optimal or maladaptive responses to training which are necessary to be successful in the athletes’ respective sports. This view of sport training is very different from the perspective of exercising for health and wellness, with recommendations to perform both aerobic and resistance exercise sessions as part of a total exercise program. In this case, the goal of improving health and wellness supersedes the potential interference of performing aerobic and strength training together. When training athletes for improvements in sports performance, the specific variables that contribute to success in sport must be the priority and a coach must keep the training priorities in mind when designing and implementing training programs. If an athlete requires maximum speed, power output, or RFD, then the training program must focus on enhancing those variables and any mode of exercise that is counter to improving those variables should be excluded from the program. If a situation arises that may require short-term periods of aerobic exercise, this simulation model could be used to estimate the degree of interference of acute aerobic exercise and, subsequently allow the coach to make informed decisions on training program design. However, we suggest that coaches consider that the model described herein represents theoretical predictions and has not yet been empirically validated.

## Data Availability

The original contributions presented in the study are included in the article/supplementary material. Further inquiries can be directed to the corresponding author.
